# Kazakhstani HER2 breast cancer digital image dataset: The ADEL dataset

**DOI:** 10.1016/j.dib.2025.112052

**Published:** 2025-09-11

**Authors:** Gauhar Dunenova, Aidos Sarsembayev, Alexandr Ivankov, Dilyara Kaidarova, Zhanna Kalmatayeva, Elvira Satbayeva, Natalya Glushkova

**Affiliations:** a Al-Farabi Kazakh National University, Almaty 050040, Kazakhstan; b School of Digital Technologies, Almaty Management University, Almaty 050060, Kazakhstan; c Independent Researcher, Almaty, 050000, Kazakhstan; d Rector Office, Asfendiyarov Kazakh National Medical University, Almaty, 050000, Kazakhstan; e Almaty Oncology Center, Almaty, 050040, Kazakhstan; f Health Research Institute, Al-Farabi Kazakh National University, Almaty, 050040, Kazakhstan

**Keywords:** Dataset, HER2, Breast cancer, Digital images

## Abstract

Breast cancer remains a leading cause of cancer-related mortality among women worldwide, with HER2-positive subtypes requiring precise diagnostic approaches to guide targeted therapy. Digital pathology and AI-based tools offer promising solutions, but their development relies heavily on high-quality digital datasets, labelled or annotated. In this study, we present a dataset of digital images of breast cancer tissue samples with immunohistochemical expression of human epidermal growth factor receptor 2 (HER2) classes 0, 1+, 2+, and 3+. Breast cancer tissue samples were formalin-fixed and paraffin-embedded (FFPE), followed by the preparation of paraffin blocks and 5-µm sections. Immunohistochemical staining was performed using a Ventana Benchmark Ultra automated immunostainer with PATHWAY anti-HER2/neu (4B5) rabbit monoclonal antibodies and ULTRA VIEW detection system.

Digital images were acquired via a fully automated digital system (KFB PRO 120 scanner) at INVIVO LLP with 40x magnification and one focusing layer, ranging in size from 50 MB to 2 GB, depending on the size of the tissue sample fixed on the original slide. The dataset consists of 418 subfolders with images, each corresponding to a source image and containing a different number of tiles depending on the size of the source image. The original images were preprocessed using a conversion script that transformed SVS files into sub-images with a 1:1 aspect ratio in JPEG format. A non-overlapping sliding window approach was applied to generate these sub-images, optimized for machine learning applications.

A square window of 1000 × 1000 pixels was used to crop sub-images with a 1:1 aspect ratio. The stride of the sliding window was set to a value that was a multiple of the image resolution (as determined during preprocessing). As a result, a variable number of sub-images were generated from each original SVS image, depending on its size. The output file format was JPEG.

Clinical labeling of the data was provided by reference laboratory pathologists with expertise in advanced oncological morphology evaluations.

This dataset allows training and validation of machine learning models for the diagnosis, recognition, and classification of breast cancer using the available labeling, as well as for educational purposes for residents and pathologists.

Specifications Table

**Table:** 

Subject	Health Sciences, Medical Sciences & Pharmacology
Specific subject area	Medical Imaging in Digital Pathology of Breast Cancer and HER2 Digital Image Analysis
Type of data	Processed
Data collection	Formalin-fixed paraffin-embedded (FFPE) breast cancer tissue samples were used, with paraffin blocks prepared and 5-µm sections sectioned. The samples were stained using a Ventana Benchmark Ultra automated immunostainer with PATHWAY anti-HER2/neu (4B5) rabbit monoclonal antibody and the ULTRA VIEW detection system.
Data source location	The immunohistochemically stained glass slides of HER2 from breast cancer patients diagnosed between 2022 and 2024 were collected from the archives of the Immunohistochemistry and Molecular Diagnostics Laboratory at the Almaty Oncology Center and the Center for Morphological Diagnostics at the Kazakh Institute of Oncology, Almaty, Kazakhstan
Data accessibility	The images are available online on Huggingface (in **JPEG** format) and the Zenodo website (in **PNG** format) Repository name: HER2 Breast Cancer Digital Image Dataset (ADEL Dataset). Data identification number: https://zenodo.org/records/15872690 Direct URL to data: https://huggingface.co/datasets/aidosSarsembayev/adel_dataset_1
Related research article	None

## 1 Value of the Data

• This dataset provides a collection of digitalized immunohistochemistry images of breast cancer tissues expressing HER2 at grades 0, 1+, 2+, and 3+. The combination of expert pathological labeling and high-resolution image acquisition makes it a robust resource for the development and benchmarking of artificial intelligence and machine learning algorithms in digital pathology.
• The dataset offers a comprehensive and clinically relevant distribution of HER2 scores, including detailed ISH status for equivocal cases, providing valuable material for training and validating AI models in HER2 assessment, including HER2-low cases.
• These data fill the gap in publicly available, labeled clinical datasets, which is particularly valuable for improving diagnostic accuracy, reducing interobserver variability, supporting AI-powered HER2 scoring systems, and making a positive contribution to bringing automated diagnostics of routine processes closer.
• The clinical dataset can be used for training for educational purposes for residents and pathologists.

## 2 Background

Breast cancer (BC) is the leading cancer in the world population, with >2.3 million new cases and 700 thousand deaths annually [1]. The incidence of BC is predicted to rise to 3 million cases with mortality reaching 1 million by 2040 [2]. BC incidence exceeds 80 per 100,000 women in high-income countries (e.g., Australia, Europe, North America) and is below 40 in Central America, Africa, and parts of Asia [3]. Five-year survival rates exceed 90 % in these countries, compared to 66 % in India and 40 % in South Africa (WHO). In Kazakhstan, BC ranks first among cancers in women: in 2022, 4570 new cases and 1574 deaths were registered, with age-standardized incidence and mortality rates of 36.9 and 12.3 per 100,000, respectively [4]. The five-year overall survival rate is 57.1 %. HER2 (human epidermal growth factor receptor 2) expression is crucial for BC prognosis, survival, and treatment strategies [5,6]. Immunohistochemical (IHC) screening of HER2 became a routine procedure for BC patients in the 1990s following the introduction of HER2-targeted therapies such as trastuzumab [[7], [8], [9]].

Manual IHC assessment of HER2 is a complex and time-consuming process that requires a high level of specialist training [10]. Today, artificial intelligence (AI) methods are increasingly being used to complement the expertise of pathologists, offering the potential for more consistent diagnostic accuracy. Digital pathology and machine learning provide new opportunities for automating HER2 assessment [11], demonstrating high accuracy in experimental studies [12,13]. Several publicly available HER2 datasets are used to train and evaluate machine learning algorithms, including the dataset hosted by the University of Warwick [14] and the ITU-MED-1 and ITU-MED-2 datasets [15]. In general, publicly available datasets address key issues of accessibility, labeling, and experimental reproducibility. However, public datasets may not fully capture real-world clinical challenges, such as variability in image quality and quantity. As a result, many recent studies increasingly rely on clinical datasets when testing HER2 digital analysis algorithms [16]. To contribute to advancing automated HER2 assessment, we aimed to create the first Kazakhstani HER2 digital image dataset for breast cancer.

## 3 Data Description

The dataset hosted on the Hugging Face platform comprises 43 main archives containing tiles from 418 digital scans of BC tissue stained for IHC HER2 expression.

Each main archive (e.g., HER2_001_009.tar.gz) contains subfolders, with each subfolder holding tiles derived from a single original HER2-stained image. For example, the subfolder named HER2_001 contains 9 tiles, labeled sequentially (e.g., HER2_001_00001.jpg).

A CSV file named labels.csv, located at the path [HER2 Breast Cancer Digital Image Dataset (ADEL Dataset)], provides the corresponding HER2 scores for each image (subfolder). Each entry in the CSV file matches an image filename with its respective HER2 score. These scores were extracted from diagnostic pathology reports and verified by a board-certified pathologist to ensure accuracy and consistency.

## 4 Experimental Design, Materials and Methods

### 4.1 Patient samples

The IHC-stained glass slides of HER2 from patients with BC diagnosed between 2022–2024 were collected from the archives of the Immunohistochemistry and Molecular Diagnostics Laboratory at the Almaty Oncology Center and the Center for Morphological Diagnostics at the Kazakh Institute of Oncology. The glass slides contained both primary and metastatic tumors, primarily biopsies with fewer surgical samples.

In addition to the slides, we obtained the pathological reports of the patients with HER2 assessment, which included scores (0, 1+, 2+, 3+) and were interpreted as positive, negative, or equivocal results. In the case of an equivocal score (2+), the pathological report was supported with in situ hybridization amplification test results.

All included slides were evaluated for technical adequacy and examined for the presence of any evident invasive carcinoma. The study complied with the Declaration of Helsinki and was approved by the Institutional Review Board of al-Farabi Kazakh National University (No. IRB-A310 dated 05/20/2021) with the approval extension accepted in 2024. The study was conducted using fully anonymized immunohistochemical digital images derived from archived human BC tissue samples. All data were obtained retrospectively from existing institutional archives and did not involve any direct interaction with human subjects or the collection of new specimens. Although the images were fully anonymized and contained no personally identifiable information, they were derived from human breast cancer tissue. Therefore, ethical approval was nevertheless obtained from the Institutional Review Board to ensure compliance with institutional and international standards. The IRB waived the requirement for informed consent.

### 4.2 HER2 IHC staining

The HER2-stained slides were prepared from 5 µm sections of the original formalin-fixed paraffin-embedded tumor samples and stained according to the manufacturer’s instructions using an automated immunostainer (Ventana, Benchmark Ultra) with PATHWAY anti-HER2/neu (4B5) rabbit monoclonal primary antibodies (Ventana) and ULTRA VIEW systemic detection. To ensure staining quality, internal laboratory controls were used: the negative control consisted of a section from the block under study with Rabbit Monoclonal Negative Control Ig applied, while the positive control was a section from a tumor block with a confirmed HER2 status verified by the ISH method.

### 4.3 HER2 IHC scoring

The IHC expression of HER2 was assessed and scored according to the 2018 ASCO/CAP (American Society of Clinical Oncology / College of American Pathologists) guidelines [17]. IHC reports, based on manual HER2 assessments, were retrieved from the archives of the Immunohistochemistry and Molecular Diagnostics Laboratory at the Almaty Oncology Center and the Center for Morphological Diagnostics at the Kazakh Institute of Oncology, both of which are national reference laboratories.

HER2 IHC scoring encompasses three key criteria: staining intensity, circumferential membrane staining pattern, and the proportion of positive cells. Cases with no staining or faint, barely perceptible membrane staining in <10 % of invasive tumor cells are classified as 0 (HER2-negative). When faint or barely noticeable membrane staining is observed in >10 % of invasive tumor cells, the case is reported as 1+ (HER2-negative). Cases are classified as 2+ (HER2-equivocal) if fewer than 10 % of invasive cancer cells show complete membrane staining or if >10 % of tumor cells display weak to moderate membranous staining.

If >10 % of cancer cells exhibited strong, complete membrane staining, the case is classified as 3+ and considered HER2 positive.

HER2-equivocal (2+) cases require additional in situ hybridization (ISH) testing. HER2 gene amplification in the nucleus is evaluated via ISH, most commonly fluorescence in situ hybridization (FISH), although chromogenic ISH (CISH) and silver-enhanced ISH (SISH) are also used. HER2 gene amplification is a key diagnostic criterion and is defined as a HER2 gene-to-chromosome 17 (HER2/CEP17) ratio of at least 2.0 by FISH or by HER2 copy number analysis.

According to the ASCO/CAP guidelines, HER2 expression with an IHC level of 1+ or 2+ with a negative ISH result is currently classified as HER2-low status.

Table 1 shows the criteria for HER2 status scoring.

**Table 1:** HER2 status scoring.

HER2 score and interpretation	Staining patterns
IHC 0 negative	No staining or incomplete membrane staining that is faint or barely perceptible and in ≤10 % of tumor cells
IHC 1+ negative	Incomplete membrane staining that is faint or barely perceptible and in >10 % of tumor cells
IHC 2+ equivocal	Weak to moderate complete membrane staining in >10 % of tumor cells OR Complete and intense membrane staining in ≤10 % of tumor cells
IHC 3+ positive	Complete and intense staining of circumferential membrane in >10 % of tumor cells

A total of 425 images were initially scanned, of which 7 were excluded due to poor quality or the absence of reliable ground truth annotations. The final dataset comprises 418 high-quality images. Among them, the largest proportion represents cases with negative HER2 expression (“0”): 138 images. Cases with weak HER2 expression (“1+”) account for 102 images. There are 75 images with an equivocal “2+” score, representing a group of uncertain HER2 status that typically require confirmatory testing by in situ hybridization (ISH). To clarify these, ISH results were specified: 19 were ISH positive (HER2 amplification), and 45 were ISH-non-amplified. Finally, 103 images correspond to cases with high HER2 expression (“3+”) (Table 2).

**Table 2:** Distribution of digital images in the dataset by HER2 score.

HER2 assessment	n
“0”	138 (33 %)
“1+”	102 (24.4 %)
“2+”	75 (17.9 %) (19 - ISH positive, 45 -ISH negative)
“3+”	103 (24.6 %)
Total	418 (100 %)

The mean age of patients whose slides were scanned was 58.1 ± 12.2 years, with 110 patients (26.3 %) in the premenopausal age group (<50 years) and 308 patients (73.7 %) in the ≥50 years age group. Of the known 399 histological types, 92.7 % were nonspecific carcinomas, predominantly invasive ductal carcinomas. Grade 2 malignancy (281/366), hormone-positive BC (297/410), and a high proliferative index (310/410) were most prevalent.

### 4.4 HER2 IHC scanning

The original HER2 slides were scanned using the fully automated KFB PRO 120 digital pathology system at the INVIVO LLP laboratory, with 40x magnification and a single focal layer. The resulting image files were saved in SVS format on the lead author's hard drive with encrypted access. The file sizes of the digital slides ranged from 50 MB to 2 GB, depending on the size of the biopsy material on the original glass slide.

### 4.5 Image preprocessing

Due to the substantial file size of SVS images, conversion to lighter formats was necessary to facilitate experimental work, particularly since the primary goal of the dataset is its application in AI algorithm development. A non-overlapping sliding window approach was applied to each image, moving from left to right and top to bottom. A square window of 1000 × 1000 pixels was used to crop sub-images with a 1:1 aspect ratio. The stride of the sliding window was set to a value that was a multiple of the image resolution, as determined during preprocessing. As a result, a variable number of sub-images were generated from each original SVS image, depending on its size. The output file format was JPEG.

The custom conversion script for restoring (“stitching”) the original image from JPEG tiles is available on GitHub, a web-based platform for hosting IT projects (https://github.com/asarsembayev/her2_data_processing). End users can compress the reconstructed images to PNG format or divide them into smaller tiles for processing.

Because the “sliced” images are indexed in a strict order, the restoration script accurately reassembles the full image without any loss of spatial relationships between image elements or quality.

**Fig. 1:**
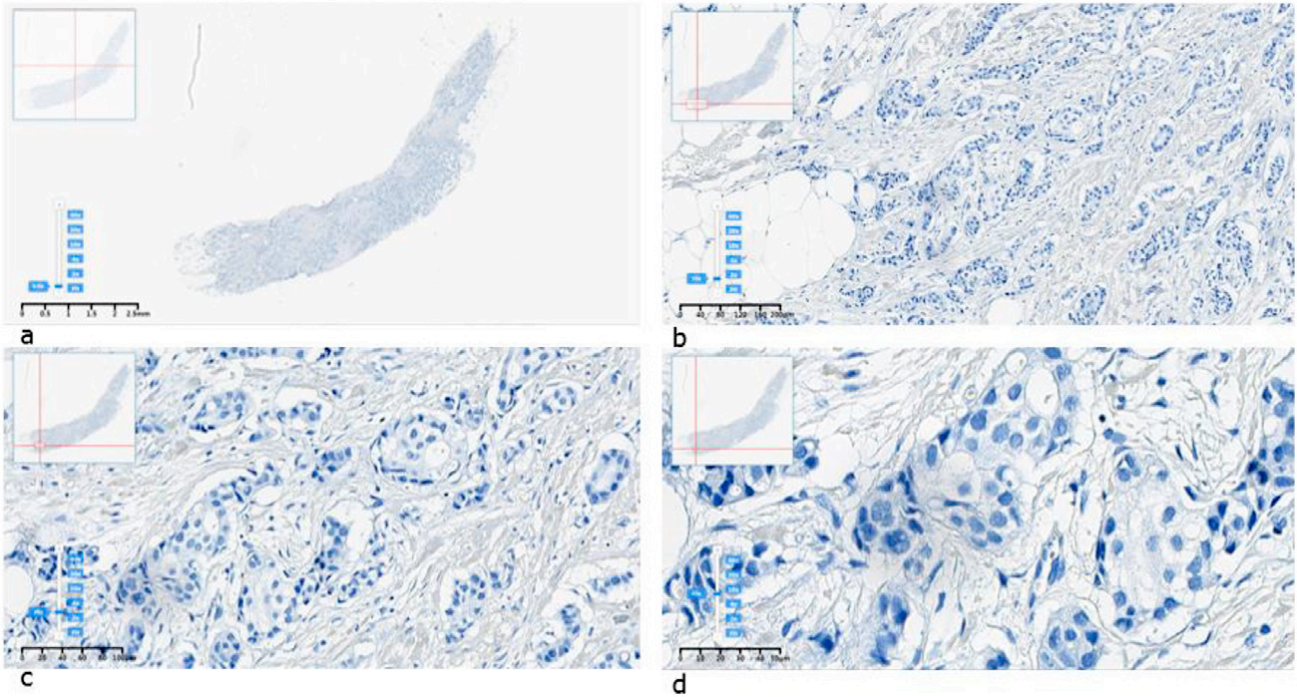
Example of an IHC HER2 “0” image labeled by pathologists in SVS format: a) original, b) x10, c) x20, d) x40 resolution.

**Fig. 2:**
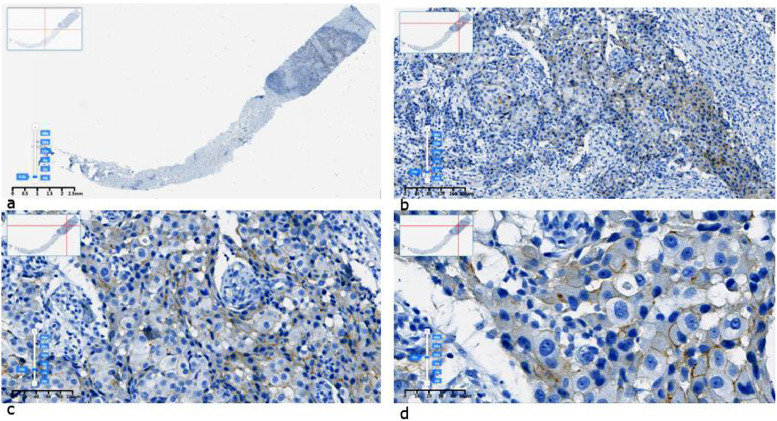
Example of an IHC HER2 “1+” image labeled by pathologists in SVS format: a) original, b) × 10, c) × 20, d) × 40 resolution.

**Fig. 3:**
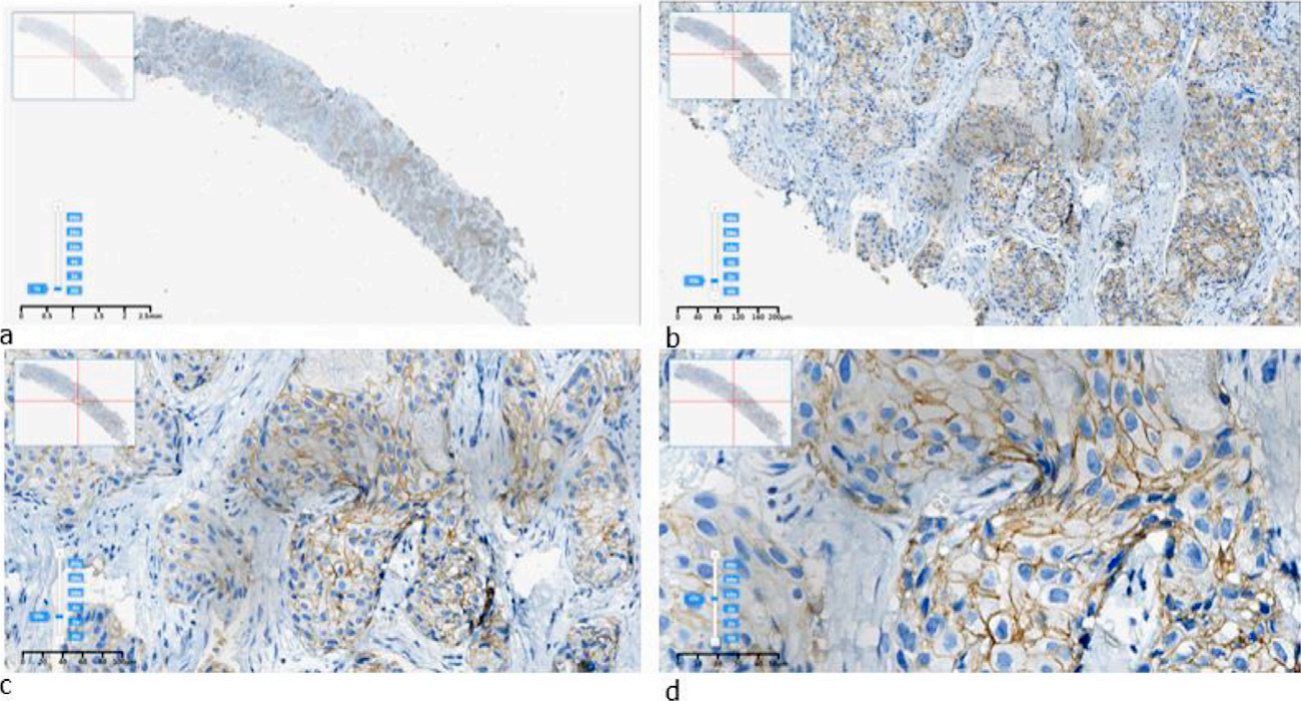
Example of an IHC HER2 “2+” image labeled by pathologists in SVS format: a) original, b) × 10, c) × 20, d) × 40 resolution.

**Fig. 4:**
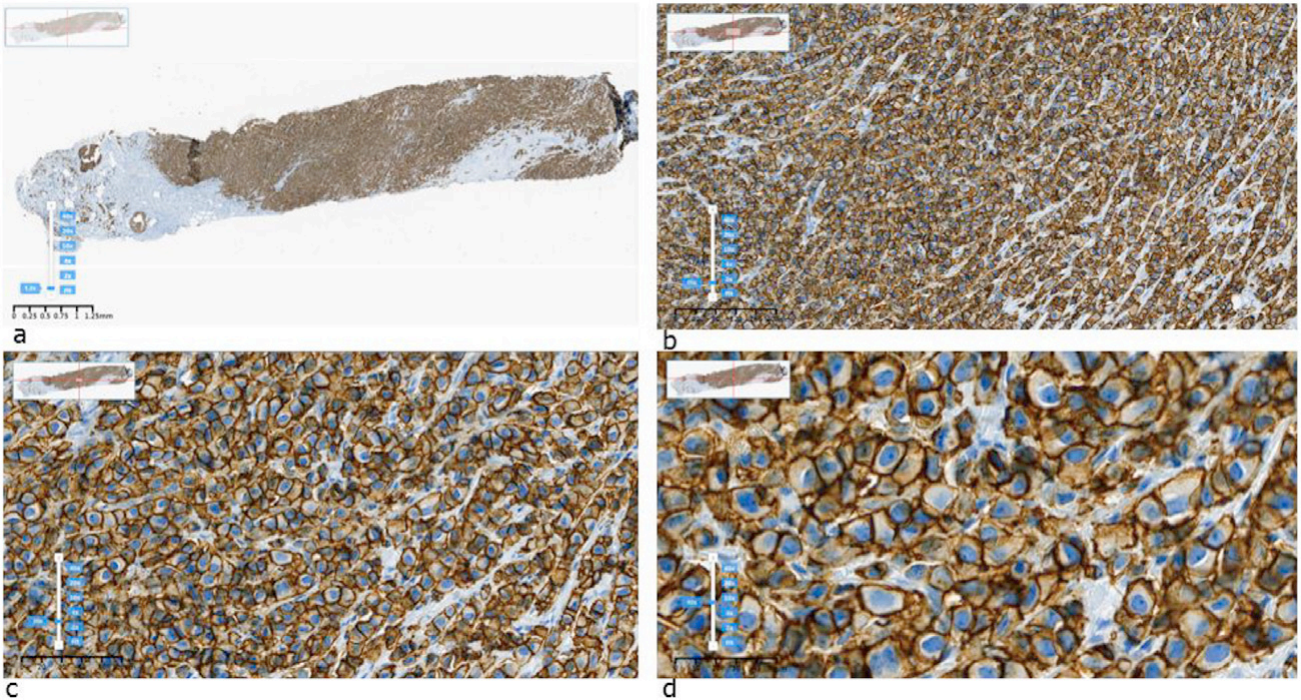
Example of an IHC HER2 “3+” image labeled by pathologists in SVS format: a) original, b) × 10, c) × 20, d) × 40 resolution.

Fig. 1, Fig. 2, Fig. 3, Fig. 4 show representative images of the dataset of all HER2 scoring classes (0, 1+, 2+, and 3+).

## Limitations

During data collection, we encountered the following limitations. First, the dataset is imbalanced, particularly in the low HER2 score categories (1+ and 2+ ISH non-amplified), which are clinically significant for automation and second-opinion assessment. Second, the dataset is currently labeled, but it has not yet been annotated.

In future work, we plan to enhance the analysis by implementing image patching techniques and conducting an internal review of the images as part of quality control measures. This approach will allow for more detailed feature extraction and may improve the classification performance of our model.

## Ethics Statement

All the authors confirm that we have read and adhered to the ethical requirements for publication in *Data in Brief****.*** The current study involved IHC images derived from human breast cancer tissue. However, as the images were fully anonymized, contained no personally identifiable information, and were obtained retrospectively from existing archives without any direct interaction with patients, the study does not meet the definition of research involving human subjects according to applicable ethical guidelines.

All the authors also confirm that the current work does not involve animal experiments or any data collected from social media platforms.

Furthermore, all the authors consciously confirm the following:

- All the authors contributed equally to this work.
- This article is an original paper and has not been previously published, except as part of the first author's academic thesis in the Russian language within the local academic system [18].
- The article is not under consideration for publication elsewhere.
- The publication of the article has been approved by all the authors.
- If accepted, the article will not be published elsewhere in the same form, in English or in any other language, including electronically, without the written consent of the copyright holder.

## Credit author statement

**Gauhar Dunenova, Natalya Glushkova**: Conceptualization, Writing – original draft; **Natalya Glushkova**: Project administration; **Natalya Glushkova, Alexandr Ivankov, Zhanna Kalmatayeva, Dilyara Kaidarova**: Writing – review & editing, Supervision; **Aidos Sarsembayev**: Conceptualization, Data curation; **Elvira Satbayeva**: Resources, Data Curation.

## Data Availability

HuggingfaceHER2 Breast Cancer Digital Image Dataset (ADEL Dataset) (Original data)
